# Investigating the Relationship between Media Usage, Depression, and Quality of Life among Older Adults

**DOI:** 10.3390/healthcare9091154

**Published:** 2021-09-03

**Authors:** Kuan-Ting Wang, Shih-Hau Fu, Pei-Lun Hsieh, Ying-Lien Lin, Shang-Yu Yang

**Affiliations:** 1 Department of Healthcare Administration, College of Medical and Health Science, Asia University, Taichung 41354, Taiwan; theccww@gmail.com; 2 Department of Acupressure Technology, Chung Hwa University of Medical Technology, Tainan 717, Taiwan; ptchrisman@gmail.com; 3 Department of Nursing, College of Health, National Taichung University of Science and Technology, Taichung 40343, Taiwan; peilun914@gmail.com; 4 Department of Industrial and Information Management, National Cheng Kung University, Tainan 701, Taiwan; r38021019@gs.ncku.edu.tw

**Keywords:** media usage, depression, quality of life, older adults

## Abstract

Background: The uses and gratifications theory suggests that various types of media can greatly affect people’s lives. This is especially true among older adults who tend to use media for leisure. However, there is insufficient research regarding the relationship between media usage, depression, and quality of life among older adults. Purpose: The purpose of this study was to explore the association between media usage (traditional and internet media), depression, and quality of life among older adults. Methods: Using a cross-sectional design, this study recruited individuals aged 65 years and older in central Taiwan and collected data via a structured questionnaire. Contents of the questionnaire included demographic details, a media usage behavior questionnaire, the Brief Symptoms Rating Scale (BSRS-5), and the Quality of Life Scale developed by the World Health Organization (WHOQOL-BREF). Subsequently, multiple regression analyses were conducted to investigate the association between media usage, depression, and quality of life of older adults. Results: The average age of the 252 participants (107 males) was 70.0 ± 5.4 years. Regression analysis revealed a significant, negative relationship between the number of hours spent watching television and the degree of depression. Additionally, the number of hours spent watching television was significantly negatively related to the quality of life in terms of both the psychological health and social relationships categories. In contrast, the number of hours spent reading newspapers and magazines was significantly positively related to quality of life in the categories of psychological health, social relationships, and environment. Finally, the number of hours spent browsing internet media was significantly positively related to the quality of life in the psychological health category. Conclusion: Media usage may affect the degree of depression and quality of life of older adults depending on the type of media and amount of usage.

## 1. Introduction

Media has evolved significantly with changing times and the advancement of technology. Based on its complex message delivery system and continuously innovative characteristics, media is divided into two types: (1) non-internet media, which includes traditional media such as television (TV), newspapers, magazines, and radio, and (2) internet media, which includes novel online media such as the internet and social networking sites [1,2,3,4]. Both these media types can influence the mood and behavior of the audience (readers and listeners), reflect their individualized thoughts and preferences, and consequently affect their quality of life [5]. Having a gradually shrinking social circle (due to declined physiological function or financial ability), older people tend to use media (such as watching TV) as their primary source of daily information and entertainment [6]. However, previous research suggests that the mental health of the audience is dependent on media usage [7]. In other words, the duration of media usage and the type of the media consumed may affect the mental health and quality of life of older adults [8,9].

Many older people have established the habit of using non-internet media (such as watching TV, reading newspapers and magazines, and listening to the radio) and believe that modern communication technologies (such as e-books) cannot replace printed newspapers and magazines [10]. In Taiwan, among the different types of non-internet media usage, people spend the most time watching TV. People aged between 60 and 69 years spend an average of 6.20 days per week watching TV. This increases to 6.37 days for people aged over 70 years [11]. In addition, the average number of hours spent watching TV per week increases with age, reaching the highest of 24.51 h among people aged 66 years and older. In other words, increasing audience age is associated with more frequent and longer TV-watching [12]. The novel coronavirus disease 2019 (COVID-19) pandemic forced many older people to stay at home; therefore, they relied on watching TV, reading magazines, or listening to the radio for entertainment. Although research has suggested that long hours of watching TV or reading magazines can cause depression [13], there is insufficient research on the relationship between the usage of non-internet media and depression. Therefore, further exploration is required to fully understand the relationship between the two variables.

Compared to non-internet media, internet media is becoming increasingly popular among older adults due to its high interactivity and powerful search capabilities. In Taiwan, the average internet usage rate of people aged over 60 years and those aged over 65 years (inclusive) is 60.2% and 42.8%, respectively. Compared to past statistics, not only has the number of users increased substantially, but the hours of social media usage have also increased considerably [14]. Some studies have indicated that there is a significant positive correlation between the frequency of internet media usage and negative emotions among older adults [15,16]. However, others [9] have proposed the opposite, that is, access to internet media can reduce an individual’s negative emotions. Furthermore, a different study has reported no correlation between users’ internet media usage and their loneliness as well as mental health [8]. It is, therefore, clear that existing research on the relationship between internet media usage and mental health among older adults has failed to reach a consensus.

Media usage may affect different aspects of quality of life among older adults (such as happiness and life satisfaction) [17], although these subjective feelings are key indicators of an individual’s quality of life. Therefore, it has been concluded that media not only provides information and entertainment, but also indirectly reflects both the mental status and quality of life of older adults [18]. Furthermore, as the social circle of older adults becomes smaller with increasing age, the effect of media information is magnified in certain circumstances, as it becomes older adults’ only access to the world. This, in turn, influences their quality of life [19].

The uses and gratifications theory conceptualizes the idea that people use media to gratify specific wants and needs; therefore, the theory proposes that various types of media can greatly affect people’s lives [20,21,22]. This is true especially among older adults, as they tend to use media for leisure [20,21,22]. However, there is insufficient research on the correlation between media usage, depression, and quality of life among older adults. A better understanding of this relationship could improve our knowledge of the possible impact of media usage on older adults. The purpose of this study was to explore media usage (both non-internet and internet media) among older adults and investigate the association between media usage, depression, and quality of life. The results of this study could provide a better understanding of the possible impact of different types of media and their usage on the mental health and the quality of life of older adults.

## 2. Methods

### 2.1. Study Design and Subject Recruitment

Using a cross-sectional design, this study collected data from individuals belonging to a certain community in Taichung City, Taiwan, via a structured questionnaire. Data collection spanned from January 2021 to April 2021. Research assistants approached different community organizations (including community bases, activity centers, etc.) in order to explain the research aims and recruit participants. Once written consent was given, participants were taken to a quiet place to complete the questionnaire. The inclusion criteria were as follows: (1) Taiwanese senior citizens above 65 years of age, (2) capable of communicating in Mandarin and understanding the contents of the questionnaire, and (3) having no disruption in consciousness and no obvious cognitive dysfunction. Additionally, the exclusion criteria were: (1) a previous history of mental illness and (2) an inability to understand the contents of the questionnaire or failure to complete the questionnaire. The study was reviewed and approved by the Human Research Ethics Committee of the Jen-ai Hospital (case number 110-15). A total of 254 participants agreed to participate in the study. Two responses were excluded upon review due to missing data, resulting in a total of 252 valid and complete responses.

### 2.2. Questionnaire

The questionnaire consisted of five parts. The first part contained items relating to demographic information, namely gender, age, education level, marital status, monthly income or disposable income, residential status, number of chronic diseases, and employment status. The second part contained items regarding media usage behavior of older adults. Based on a previous study [23], this study designed a “media usage behavior questionnaire,” the validity of which was verified by three experts (i.e., an assistant professor in the Department of Healthcare Administration Management, an assistant professor in the Department of Nursing, and an assistant professor in the Department of Occupational Therapy). With the purpose of exploring the daily media usage behavior of older adults, the questionnaire covered four types of media, namely TV, newspapers and magazines, radio, and internet media. Topics covered included participants’ average daily media usage hours during weekdays and weekends, the motivation behind using media, the location of using media, the most frequently used electronic device, etc.

The third part of the questionnaire surveyed participants’ degree of depression. The Brief Symptoms Rating Scale (BSRS-5) was used for this purpose. Designed by Lee et al. [24], the scale is self-administered and consists of 5 questions. It has been widely used in community screening due to the limited number of questions and the ease of completion [24]. BSRS-5 was scored on a 5-point Likert scale, with 0 representing “Not at all”, 1 representing “A little”, 2 representing “Moderately”, 3 representing “Quite a bit”, and 4 representing “Extremely.” The higher the score, the more depressed the participant. The total score could range between 0 and 20 and could be divided into four categories, with 0–6 indicating a normal range in which the subject is well adapted both physically and mentally, 6–9 indicating mild emotional distress, 10–14 indicating moderate emotional distress, and 15 and beyond indicating severe emotional distress. Both the reliability and validity of BSRS-5 have been well verified [25,26]. In this study, the Cronbach’s alpha of BSRS-5 was 0.84.

The fourth part contained items relating to the quality of life of older adults. This study adopted the Taiwanese version of the Abbreviated World Health Organization Quality of Life (WHOQOL-BREF) to measure participants’ quality of life. The questionnaire is self-administered and consists of 28 questions. Its contents cover a total of four categories of quality of life, including physical health (7 questions), psychological health (6 questions), social relationships (4 questions), and environment (9 questions). WHOQOL-BREF is scored on the 5-point Likert scale, with 1 being the lowest score of each question and 5 being the highest. The higher the score, the higher the subject’s quality of life. Both the reliability and validity of WHOQOL-BREF have been well verified [27,28]. In this study, the Cronbach’s alpha of the physical health, psychological health, social relationships, and environment categories were 0.82, 0.83, 0.78, and 0.83, respectively.

### 2.3. Statistical Analysis

In this study, the SPSS 25.0 version for Mac (IBM, Armonk, New York) was used for data analysis. Descriptive statistics were first analyzed to understand the demographic data, results of the media usage behavior questionnaire, and scores of the BSRS-5 and WHOQOL-BREF. Subsequently, based on the findings of a previous study [29], subjects were divided into general audiences and engaged audiences based on their hours of media usage. The Student’s *t*-test was then used to test whether there were significant differences in the scores of BSRS-5 and the four categories of WHOQOL-BREF between these two types of audiences. The threshold between general and engaged audiences was 3 h for the number of hours accessing TV and internet media, and 1 h for the number of hours accessing newspapers and magazines [29]. 

Multiple regression analysis was used to investigate the relationship between media usage and depression. The BSRS-5 score was introduced as the dependent variable of the multiple regression model and the average daily media usage hours (including via TV, newspapers and magazines, radio, and internet media) was introduced as the independent variable. At the same time, all the demographic variables were controlled for, as previous literature suggested that they could affect both the independent and dependent variables of this study. These demographic variables included gender [30], age [31], educational level [32], marital status [31], monthly or disposable income [33], residential status [34], number of chronic diseases [35], and employment status [36].

Similarly, multiple regression analysis was used to investigate the relationship between media usage and quality of life. Eight multiple regression analyses were run, each with one of the four categories of the WHOQOL-BREF as the dependent variable and media usage during either weekdays or weekends as the independent variables. At the same time, all the demographic variables were controlled for, as previous literature suggested that they could affect both the independent and dependent variables of this study. These demographic variables included gender [28], age [37], education level [38], marital status [28], monthly or disposable income [37], residential status [39], number of chronic diseases [40], and employment status [41]. In addition, after collinearity diagnostics were run on different multiple regression models, it was found that the variance inflation factor (VIF) of the independent variables of all models was less than 10, indicating that the problem of collinearity could be ignored [42].

## 3. Results

### 3.1. Participant Background

A total of 252 individuals (107 male, 145 female) participated in this study. The age of participants ranged from 65 to 88 years with a mean age of 70.0 years ± 5.4 years. The demographic data of the participants are presented in Table 1. More than half of the participants had an education degree of high school and above (59.2%) were married or cohabiting (69.4%), had a monthly or disposable income of more than NT$ 20,000 (60.7%), were living together with relatives or friends (74.6%), and did not have a full-time job (80.6%). On average, participants scored 3.29 ± 3.15 on the BSRS-5. Additionally, participants scored 14.58 ± 2.06, 13.63 ± 2.40, 14.10 ± 2.27, and 14.53 ± 1.88 in the physical health, psychological health, social relationships, and environment categories of the WHOQOL-BREF, respectively.

Participants’ average media usage hours during both weekdays and weekends are displayed in Table 2. During weekdays, a majority of participants spent more than 4 h per day watching TV (23.0%), less than 1 h reading newspapers and magazines (74.2%), less than 1 h listening to the radio (63.5%), and 2–3 h browsing internet media (29.9%). Similarly, during weekends, a majority of participants spent more than 4 h per day watching TV (27.0%), less than 1 h reading newspapers and magazines (75.0%), less than 1 h listening to the radio (68.3%), and 2–3 h browsing internet media (32.4%). In addition, participants’ motivations for watching TV were mostly to obtain information (35.0%), followed by entertainment (24.6%) and to kill time (15.1%), and the venue was primarily their homes (69.3%), followed by community activity centers (10.3%) and friends’ houses (8.0%). Alternatively, motivations for reading newspapers and magazines were mostly to obtain information (41.0%), followed by entertainment (22.0%) and to kill time (16.8%), which occurred primarily in their homes (66.0%), followed by community activity centers (15.6%) and friends’ houses (6.1%). Similarly, in terms of listening to the radio, motivations were predominantly to obtain information (41.2%), followed by entertainment (21.2%) and to kill time (20.4%), and the venue was primarily their homes (53.8%), followed by vehicles (24.3%) and community activity centers (6.2%). Lastly, participants browsed internet media mostly to obtain information (32.0%), followed by a need to satisfy social needs (28.0%) and entertainment (18.4%). The most frequently used devices were smartphones (32.0%), followed by tablets (5.4%) and computers (2.5%).

### 3.2. Differences between Different Levels of Media Users in the BSRS-5 and WHOQOL-BREF

The results of the Student’s *t*-test are presented in Table 3. In terms of watching TV, the BSRS-5 score of participants watching TV for more than 3 h per day over the weekend was significantly lower (*p* < 0.05) than the score of those watching for less than 3 h. In terms of reading newspapers and magazines, the scores of participants reading newspapers and magazines for more than 1 h during weekdays in all four categories of the WHOQOL-BREF were significantly higher (*p* < 0.05) than those of participants reading less. In terms of listening to the radio, the scores of participants listening to the radio for more than 1 h over the weekend in three categories of the WHOQOL-BREF (all categories other than environment) were significantly lower *p* < 0.05) than the scores of participants listening less. Lastly, in terms of browsing internet media, the scores of participants browsing internet media for more than 3 h during weekdays were significantly higher (*p* < 0.05) than the scores of participants browsing less in the psychological health category of the WHOQOL-BREF. In addition, scores of both the physical health and psychological health categories of the WHOQOL-BREF of participants browsing internet media for more than 3 h during weekdays were significantly higher (*p* < 0.05) than the scores of participants browsing less. 

### 3.3. Association between Media Usage, BSRS-5, and WHOQOL-BREF

Table 4 presents the results of two multiple regression analyses in which demographic parameters were controlled for and BSRS-5 scores were predicted on the basis of average daily media usage hours during weekdays and weekends. During weekdays, media usage hours of none of the four media types were significant predictors of the total score of the BSRS-5. Over the weekend, only the media usage hours of TV was a significant predictor of the total score of the BSRS-5 (B: −0.35, SE: 0.13, *p* < 0.01).

Table 5 shows the results of the multiple regression analyses in which demographic parameters are controlled for and the WHOQOL-BREF category scores are predicted on the basis of average daily media usage hours during weekdays and weekends. During weekdays, both the number of hours spent watching TV (B: −0.25, SE: 0.10, *p* < 0.01) and the number of hours spent reading newspapers and magazines (B: 0.45, SE: 0.14, *p* < 0.01) were significant predictors of psychological health. In addition, the number of hours spent reading newspapers and magazines was also a significant predictor of both social relationships (B: 0.38, SE: 0.14, *p* < 0.01) and environment (B: 0.27, SE: 0.12, *p* < 0.05). Alternatively, over the weekend, the number of hours spent watching TV (B: −0.25, SE: 0.09, *p* < 0.01), reading newspapers and magazines (B: 0.41, SE: 0.15, *p* < 0.01), and browsing internet media (B: 0.17, SE: 0.08, *p* < 0.05) were significant predictors of psychological health. In addition, both the number of hours spent watching TV (B: −0.24, SE: 0.09, *p* < 0.01) and reading newspapers and magazines (B: 0.38, SE: 0.14, *p* < 0.01) were significant predictors of social relationships. 

## 4. Discussion

Recent research has focused on internet media (such as social media) but has largely ignored the fact that there are several groups (such as older adults) who still use traditional media regularly [43]. In addition, the older adult population is often not included in studies related to internet media (e.g., studies on internet browsing devices such as smartphones and tablets) [43]. In contrast, ours is one of the few studies that has investigated the usage of both traditional and internet media among older adults. The results of this study show that most participants spent more than 4 h per day watching TV, both during weekdays and over the weekend. This is similar to the findings from a previous study that found that older adults aged 66 years and over, on average, spent 3.5 h watching TV each day [44]. The motivations behind media usage were predominantly “to obtain information”, followed by “entertainment” and “to kill time”, and the venue of media usage was usually at home. Therefore, it is speculated that as the majority of the participants are accustomed to using TV as their main source of news and entertainment, TV has become an integral part of their life. Contrastingly, our study found that most participants spent less than 1 h reading newspapers and magazines both during weekdays and over the weekend, which is consistent with the results from a previous study [45]. When older people read newspapers and magazines, the most frequently visited pages are the news on the front page, the social pages, and the political pages. This indicates that in addition to being a source of news, newspapers and magazines are also used as a channel for social participation and interaction by older adults [45].

Most participants spent less than 1 h per day listening to the radio both during weekdays and over the weekend, which is consistent with the results from a previous study [23]. The motivations behind this behavior were primarily “to obtain information”, followed by “entertainment” and “to kill time”, and the venue was usually at home. In today’s world, the main audience for radio comprises people aged between 13–29 years old who predominantly listen to pop music and interviews of celebrities and stars [23]. Therefore, mainstream radio programs may not be well-received by older adults (as their demand is not satisfied). In addition, the popularity of traditional radio programs has declined with the rise of internet broadcasting. These reasons may have contributed to the low usage hours of the radio among the participants in this study. Conversely, most participants spent 2–3 h per day browsing internet media both during weekdays and over the weekend, indicating that internet usage is gradually gaining popularity among older adults. Furthermore, the fact that most participants in this study were living with relatives or friends also increased the possibility of using the internet [46].

The results of this study found that a greater number of hours spent watching TV on weekends was associated with a lower degree of depression. Most weekend TV programs tend to be relaxing and entertaining. Therefore, it is possible that when a viewer watches TV for entertainment or enjoys it together with relatives and friends (as a leisure activity), the severity of depression decreases. However, our study also found that during both weekdays and weekends, a greater number of hours spent watching TV was associated with poorer quality of life (namely in the psychological health and social relationship categories). One possible explanation is that the psychological health category of the WHOQOL-BREF mainly enquires about the individual’s satisfaction with their life, spirit, and appearance. Although watching TV may help alleviate negative emotions, it is incapable of raising an individual’s spiritual satisfaction and self-affirmation. Therefore, more proactive methods, including cultivating good living habits (such as exercise) [47], thinking positively [48], and engaging in interpersonal interactions [49], may be required to enhance the psychological health of older adults. In addition, as the participant spends more time watching TV, their likelihood of interacting with others reduces, which in turn affects their social relationships [50]. However, a causal relationship cannot be confirmed due to the limitations in the research design.

Our results also show that the more hours participants spend reading newspapers and magazines, either during weekdays or over the weekend, the better their quality of life. A study by He et al. [51] verified the correlation between the habit of reading newspapers and magazines among older adults and their social activities (such as participation in religious groups). This indicates that reading newspapers and magazines can have effects on the social participation and interaction of older adults. As the most accessible reading material, newspapers not only allow older adults to obtain information and kill time, but it also serves as a medium for social participation and interpersonal interaction, thereby helping them keep pace with the evolving society and changing times [45]. Active social participation and interaction helps boost the mental health of older adults [52]. In addition, despite being a focused and individual process, reading allows an individual to increase their self-confidence and ability, learn new things, absorb health knowledge, and take part in the delight of sharing information [53,54], all of which help in maintaining and enhancing physical and mental health. This study also found that a greater number of hours spent reading newspapers and magazines during weekdays was associated with better quality of life in the environment category. This suggests that relevant information acquired by older adults through newspapers and magazines, such as public transport timetables, shopping information, information on leisure activities and medical services, etc., may improve their understanding of the living environment, which in turn enhances their quality of life in the environment category.

Participants who listened to the radio for less than 1 h over the weekend had a significantly better quality of life than those who listened for more time (see Table 3). As most participants listened to the radio in their home, it is most likely that participants who spent more time listening to the radio were not performing outdoor activities, such as exercising, shopping, travelling, and visiting friends, and were instead listening to the radio at home over the weekend. Furthermore, a previous study suggested that listening to the radio could reduce engagement in routine social activities among older adults [50]. This could explain the effect of listening to the radio on the physical health, psychological health, and social relationships categories of the Quality of Life Scale. However, regression analyses showed that the number of hours spent listening to the radio was not a significant predictor of either depression or the quality of life among older adults. This implies the declining influence of radio on older adults compared to other forms of media.

With increasing age, older people may feel excluded by society in certain aspects, such as when performing various online transactions. However, improvement of the internet and popularization of smartphones have promoted the rapid development of social media and internet media, allowing older adults to connect with relatives and friends, as well as to share living information easily via texts, voice notes, photos, or videos [51,55]. In addition, despite restricted movement (due to either physiological or geographical factors), older adults can now stay in touch with their relatives and friends with the help of social media, which in turn reduces their loneliness and social isolation, and improves their happiness [56]. Another advantage of internet media is that it can be updated in real-time and can be interactive. Not only can internet media provide the latest update on news and events during all hours of the day, but it can also display real-time feedback and discussions from online viewers (whereas traditional media transmits information unidirectionally). These advantages have led older adults to find and connect with groups with similar ideas and hobbies online, and has helped improve their self-affirmation and satisfaction. These are possible reasons to explain the relationship between the number of hours participants spent browsing internet media and their quality of life in the psychological health category. Furthermore, our study also discovered that participants who spent more than 3 h browsing the internet over the weekend had a higher quality of life in the physical health category. It is possible that older adults acquire useful information online, which in turn helps them improve their engagement in social and interpersonal interactions, as well as health activities, thereby improving their physical health [30].

There are some limitations which should be considered when interpreting the results of this study. First, as a cross-sectional study, this study could not describe or explain a causal relationship between participants’ media usage, degree of depression, and quality of life. Moreover, the quality of one’s life may also affect an older person’s media usage and their risk of depression. Second, this study adopted self-administered scales. Despite their popularity and strong design, these questionnaires could not confirm participants’ real media usage time, severity of depression, and quality of life, thereby introducing the risk of research bias (such as recall bias). Third, all the participants of this study were from the same region, further limiting the generalizability of our results. Fourth, some eligible participants might be excluded due to insufficient knowledge of using new technologies or poor economic resources, which may cause research bias. Lastly, media usage was interpreted only in terms of time in this study, whereas the content of the media was not investigated, which could be an important confounding factor. Despite these limitations, the study has provided relevant information on the media usage, depression, and quality of life of older adults. These results can help institutions and professionals better understand the impact of media usage on the physical and mental health of older adults.

## 5. Conclusions

The present study found that older adults mostly watched TV as their primary source of traditional media, with the majority doing so for over 4 h per day. Additionally, participants predominantly spent 2–3 h each day browsing internet media. The motivation behind both activities were primarily “to obtain information”, followed by “entertainment” and “to kill time”, and the venue was usually at home. In addition, it was found that a greater number of hours spent watching TV over the weekend was associated with lesser severity of depression. Additionally, the study discovered that a greater number of hours spent watching TV over the weekend was associated with a lower quality of life in the psychological health and social relationships categories. Meanwhile, a greater number of hours reading newspapers and magazines was associated with a better quality of life in the psychological health, social relationships, and environment categories. Similarly, a greater number of hours browsing internet media was associated with a better quality of life in the psychological health category. These findings suggest that media usage can have both positive and negative impacts on the severity of depression and quality of life among older adults depending on the type and hours of use. Due to the limitations of the research design of this study, it is recommended that the causal relationship between media usage, depression, and quality of life is further explored in future studies. It is also recommended that, when developing media content for older adults, associations and institutions use positive materials to reduce the risk of depression and improve their quality of life.

## Figures and Tables

**Table 1:** Demographic characteristics of the participants (N = 252).

Demographic Characteristics	n	%
Gender		
Male	107	42.5%
Female	145	57.5%
Age (mean ± SD)	70.0 ± 5.4	
Education level		
Junior school (and below)	103	40.9%
High school	102	40.5%
University (and above)	47	18.7%
Marital status		
Single/divorced/separated	77	30.6%
Married /cohabiting	175	69.4%
Monthly income or disposable amount (NTD)		
<10 thousand	20	7.9%
10 thousand–20 thousand	79	31.3%
20 thousand–30 thousand	56	22.2%
30 thousand–40 thousand	51	20.2%
≧40 thousand	46	18.3%
Residential status		
Lives with relatives	188	74.6%
Lives alone	64	25.4%
Number of chronic diseases (mean ± SD)	1.3 ± 1.3	
Employment status		
YES	49	19.4%
NO	203	80.6%

Abbreviations: SD, standard deviation and NTD, New Taiwanese Dollar.

**Table 2:** Media usage of the participants.

	TV			Newspapers and Magazines			Radio			Internet Media		
Category	Hours of Use	n	%	Hours of Use	n	%	Hours of Use	n	%	Hours of Use	n	%
On weekdays	1<	38	15.1%	1<	187	74.2%	1<	160	63.5%	1<	63	26.1%
	1–2	50	19.8%	1–2	44	17.5%	1–2	45	17.9%	1–2	60	24.9%
	2–3	51	20.2%	2–3	4	1.6%	2–3	19	7.5%	2–3	72	29.9%
	3–4	55	21.8%	3–4	11	4.4%	3–4	12	4.8%	3–4	16	6.6%
	≥4	58	23.0%	≥4	6	2.4%	≥4	16	6.3%	≥4	30	12.4%
On weekends	1<	45	17.9%	1<	189	75.0%	1<	172	68.3%	1<	66	27.4%
	1–2	52	20.6%	1–2	39	15.5%	1–2	42	16.7%	1–2	53	22.0%
	2–3	41	16.3%	2–3	8	3.2%	2–3	19	7.5%	2–3	78	32.4%
	3–4	46	18.3%	3–4	10	4.0%	3–4	7	2.8%	3–4	20	8.3%
	≥4	68	27.0%	≥4	6	2.4%	≥4	12	4.8%	≥4	24	10.0%

**Table 3:** Differences between different levels of media users by hours in the BSRS-5 and WHOQOL-BREF.

	TV						Newspapers and Magazines						Radio						Internet Media					
Category	On Weekdays			On Weekends			On Weekdays			On Weekends			On Weekdays			On Weekends			On Weekdays			On Weekends		
	<3	≥3	*p*	<3	≥3	*p*	<1	≥1	*p*	<1	≥1	*p*	<1	≥1	*p*	<1	≥1	*p*	<3	≥3	*p*	<3	≥3	*p*
BSRS-5	3.47 ± 3.35	3.07 ± 2.89	0.31	3.70 ± 3.42	2.81 ± 2.73	0.02 *	3.48 ± 3.14	2.75 ± 3.15	0.11	3.36 ± 3.17	3.10 ± 3.13	0.57	3.14 ± 3.28	3.55 ±2.91	0.32	3.20 ± 3.29	3.49 ± 2.86	0.51	3.36 ± 3.04	3.05 ± 3.54	0.51	3.31 ± 3.03	3.24 ± 3.59	0.88
WHOQOL-BREF																								
Physical health	14.65 ± 2.01	14.49 ± 2.11	0.54	14.56 ± 2.09	14.61 ± 2.03	0.86	14.40 ± 2.01	15.09 ± 2.13	0.02 *	14.46 ± 2.03	14.96 ± 2.11	0.09	14.74 ± 2.07	14.31 ± 2.02	0.11	14.76 ± 2.03	14.20 ± 2.07	0.05 *	14.47 ± 2.03	14.94 ± 2.11	0.13	14.41 ± 2.01	15.19 ± 2.12	0.01 *
Psychological health	13.83 ± 2.35	13.39 ± 2.45	0.14	13.67 ± 2.35	13.59 ± 2.47	0.80	13.40 ± 2.37	14.30 ± 2.39	<0.01 *	13.50 ± 2.41	14.02 ± 2.36	0.14	13.85 ± 2.33	13.25 ± 2.49	0.05	13.92 ± 2.31	13.02 ± 2.51	<0.01 *	13.44 ± 2.37	14.29 ± 2.42	0.02 *	13.42 ± 2.33	14.40 ± 2.54	0.01 *
Social relationships	14.33 ± 2.27	13.80 ± 2.26	0.07	14.32 ± 2.20	13.82 ± 2.34	0.09	13.89 ± 2.37	14.69 ± 1.86	<0.01 *	13.98 ± 2.38	14.43 ± 1.92	0.14	14.26 ± 2.35	13.80 ± 2.11	0.12	14.31 ± 2.31	13.64 ± 2.14	0.03 *	14.05 ± 2.21	14.25 ±2.50	0.57	14.02 ± 2.25	14.38 ± 2.35	0.29
Environment	14.55 ± 1.94	14.51 ± 1.82	0.86	14.51 ± 1.92	14.55 ± 1.84	0.87	14.38 ± 1.92	14.97 ± 1.70	0.03 *	14.46 ± 1.91	14.76 ± 1.79	0.27	14.46 ± 2.00	14.66 ± 1.65	0.41	14.48 ± 2.00	14.65 ± 1.61	0.50	14.49 ± 1.91	14.68 ± 1.78	0.50	14.45 ± 1.89	14.82 ± 1.83	0.20

* *p* < 0.05.

**Table 4:** Multiple regression analysis for identifying depression significantly related to the hours of media usage on weekdays ^†^/weekends ^†^.

	Dependent Variable	BSRS-5			
Independent Variable		B	SE	OR (95% CI)	*p*
Hours of use on weekdays					
TV		−0.23	0.14	−0.51, 0.05	0.10
Newspapers and magazines		−0.39	0.21	−0.80, 0.03	0.07
Radio		0.08	0.17	−0.25, 0.41	0.63
Internet media		0.12	0.11	−0.09, 0.34	0.25
Hours of use on weekends					
TV		−0.35	0.13	−0.62, −0.09	<0.01 *
Newspapers and magazines		−0.21	0.22	−0.64, 0.21	0.32
Radio		0.03	0.19	−0.35, 0.41	0.89
Internet media		0.18	0.11	−0.03, 0.40	0.10

^†^ Controlled for gender, age, education, marital status, monthly income or disposable amount, living status, chronic diseases, and employment; * *p* < 0.05. Abbreviations: B, regression coefficient; S.E., standard error; OR, odds ratio; and CI, confidence interval.

**Table 5:** Multiple regression analysis for identifying that the WHOQOL-BREF is significantly related to the hours of media usage on weekdays ^†^/weekends ^†^.

	Dependent Variable	Physical Health				Psychological Health				Social Relationships				Environment			
Independent Variable		B	SE	OR (95% CI)	*p*	B	SE	OR (95% CI)	*p*	B	SE	OR (95% CI)	*p*	B	SE	OR (95% CI)	*p*
Hours of use on weekdays																	
TV		−0.04	0.08	−0.20, 0.12	0.62	−0.25	0.10	−0.44, −0.06	<0.01 *	−0.15	0.09	−0.34, 0.04	0.11	−0.00	0.08	−0.16, 0.15	0.97
Newspapers and magazines		0.22	0.12	−0.02, 0.46	0.07	0.45	0.14	0.17, 0.74	<0.01 *	0.38	0.14	0.10, 0.66	<0.01 *	0.27	0.12	0.04, 0.50	0.02 *
Radio		−0.08	0.10	−0.27, 0.11	0.39	−0.09	0.11	−0.31, 0.14	0.46	−0.17	0.11	−0.39, 0.05	0.13	0.04	0.09	−0.14, 0.22	0.66
Internet media		0.08	0.06	−0.04, 0.20	0.20	0.13	0.07	−0.02, 0.27	0.09	−0.04	0.07	−0.18, 0.11	0.61	0.04	0.06	−0.08, 0.15	0.55
Hours of use on weekends																	
TV		−0.09	0.08	−0.24, 0.07	0.28	−0.25	0.09	−0.43, −0.06	<0.01 *	−0.24	0.09	−0.42, −0.06	<0.01 *	−0.07	0.08	−0.22, 0.08	0.34
Newspapers and magazines		0.23	0.12	−0.01, 0.48	0.06	0.41	0.15	0.12, 0.70	<0.01 *	0.38	0.14	0.10, 0.66	<0.01 *	0.22	0.12	−0.01, 0.46	0.06
Radio		−0.09	0.11	−0.31, 0.13	0.40	−0.16	0.13	−0.42, 0.10	0.23	−0.20	0.13	−0.46, 0.06	0.12	0.13	0.11	−0.08, 0.34	0.23
Internet media		0.11	0.06	−0.02, 0.23	0.09	0.17	0.08	0.02, 0.32	0.02 *	−0.00	0.07	−0.15, 0.14	0.99	0.04	0.06	−0.08, 0.16	0.47

^†^ Controlled for gender, age, education, marital status, monthly income or disposable amount, living status, chronic diseases, employment, and the BSRS-5; * *p* < 0.05. Abbreviations: B, regression coefficient; S.E., standard error; OR, odds ratio; and CI, confidence interval.

## Data Availability

The datasets used and/or analyzed during the present study are available from the corresponding author upon reasonable request.

## References

[1] Hoskins C., McFadyen S., Finn A. (2004). Media Economics: Applying Economics to New and Traditional Media.

[2] Leong E.K., Huang X., Stanners P.-J. (1998). Comparing the effectiveness of the web site with traditional media. J. Advert. Res..

[3] Yoon S.-J., Kim J.-H. (2001). Is the Internet more effective than traditional media? Factors affecting the choice of media. J. Advert. Res..

[4] Lin C.-C. (2013). Convergence of new and old media: New media representation in traditional news. Chin. J. Commun..

[5] Lee J.H., Lee J.H., Park S.H. (2014). Leisure activity participation as predictor of quality of life in Korean urban-dwelling elderly. Occup. Ther. Int..

[6] Liao W.-C., Chiu L.-A., Yueh H.-P. (2012). A Study of Rural Elderly’s Health Information Needs and Seeking Behavior. J. Libr. Inf. Stud..

[7] Block M., Stern D.B., Raman K., Lee S., Carey J., Humphreys A.A., Mulhern F., Calder B., Schultz D., Rudick C.N. (2014). The relationship between self-report of depression and media usage. Front. Hum. Neurosci..

[8] Aarts S., Peek S.T., Wouters E.J. (2015). The relation between social network site usage and loneliness and mental health in community-dwelling older adults. Int. J. Geriatr. Psychiatry.

[9] Cotten S.R., Anderson W.A., McCullough B.M. (2013). Impact of internet use on loneliness and contact with others among older adults: Cross-sectional analysis. J. Med. Internet Res..

[10] Nimrod G. (2017). Older audiences in the digital media environment. Inf. Commun. Soc..

[11] Chang C.-C. The 2018 Taiwan Communication Survey (Phase Two, Year Two): Media Use and Social Implications. https://srda.sinica.edu.tw/datasearch_detail.php?id=3053.

[12] National Communications Commission Report on 107 Communication Market Survey Results. http://ncc.chiuanen.tw/chinese/news_detail.aspx?site_content_sn=5027&cate=0&keyword=&is_history=0&pages=0&sn_f=40971.

[13] Scherr S. (2021). Traditional media use and depression in the general population: Evidence for a non-linear relationship. Curr. Psychol..

[14] Taiwan Communication Survey A Preliminary Study on the Changes in the Use of Media by Taiwanese People-2012 to 2016. https://www.crctaiwan.nctu.edu.tw/ResultsShow_detail.asp?RS_ID=67.

[15] Hamer M., Stamatakis E. (2014). Prospective study of sedentary behavior, risk of depression, and cognitive impairment. Med. Sci. Sports Exerc..

[16] Hsiao M.-Y., Lee C.-Y., Huang H.-H., Huang Y.-D., Jiang S.-N., Chang Y.-H. (2020). Internet Use Improvement of Depression and Wellbeing in a Community of Elderly People in Central Taiwan. J. Med. Health.

[17] Thompson M.G., Heller K. (1990). Facets of support related to well-being: Quantitative social isolation and perceived family support in a sample of elderly women. Psychol. Aging.

[18] Owens D.A. (1994). Media Use Among Elderly Persons: Correlates of Life Satisfaction, Activity, Interpersonal Communication, and Public Affairs Knowledge.

[19] Spinney J.E., Scott D.M., Newbold K.B. (2009). Transport mobility benefits and quality of life: A time-use perspective of elderly Canadians. Transport. Policy.

[20] Herzog H. (1942). What Do We Really Know About Day-Time Serial Listeners?. Radio Res..

[21] Chou M.-C., Liu C.-H. (2016). Mobile instant messengers and middle-aged and elderly adults in Taiwan: Uses and gratifications. Int. J. Hum. Comput. Interact..

[22] Rubin A.M. (2009). Uses-and-gratifications perspective on media effects. Media Effects.

[23] Su C.-C., Chen W.-F. (2006). A Study on Media Usage of Different Generations: A Case Study of Eastern Integrated Consumer Profile Database 2005. J. Cyber Cult. Inf. Soc..

[24] Lee M.-B., Lee Y.-T., Yen L.-L., Lin M.-H., Lue B.-H. (1990). Reliability and Validity of Using a Brief Psychiatric Symptom Rating Scale in Clinical Practice. J. Formos Med. Assoc..

[25] Chen H.-C., Wu C.-H., Lee Y.-J., Liao S.-C., Lee M.-B. (2005). Validity of the five-item Brief Symptom Rating Scale among subjects admitted for general health screening. J. Formos Med. Assoc. Taiwan Yi Zhi.

[26] Yen J., Ko C., Yang M., Shih C., Huang W., Lian Y., Lee M. (2005). Screening depression in the community: Comparison between Taiwanese depression scale and the 5-item brief symptom Rating Scale. Taipei City Med. J..

[27] The WHOQOL-Taiwan Group (2005). The User’s Manual of the Development of the WHOQOL-BREF Taiwan Version.

[28] Yang S.-Y., Hsu D.-J., Yen C.-M., Chang J.-H. (2019). Predictive factors of life quality among packaging workers in Taiwan. Health Promot. Int..

[29] Ministry of Health and Welfare Report of the Senior Citizen Condition Survey 2017. https://www.mohw.gov.tw/dl-48636-de32ad67-19c8-46d6-b96c-8826f6039fcb.html.

[30] Yang S.-Y., Lin C.-Y., Huang Y.-C., Chang J.-H. (2018). Gender differences in the association of smartphone use with the vitality and mental health of adolescent students. J. Am. Coll. Health.

[31] Bulloch A.G., Williams J.V., Lavorato D.H., Patten S.B. (2017). The depression and marital status relationship is modified by both age and gender. J. Affect. Disord..

[32] Lopez V., Sanchez K., Killian M.O., Eghaneyan B.H. (2018). Depression screening and education: An examination of mental health literacy and stigma in a sample of Hispanic women. BMC Public Health.

[33] Patel V., Burns J.K., Dhingra M., Tarver L., Kohrt B.A., Lund C. (2018). Income inequality and depression: A systematic review and meta-analysis of the association and a scoping review of mechanisms. World Psychiatry.

[34] Honjo K., Kimura T., Baba S., Ikehara S., Kitano N., Sato T., Iso H., Kishi R., Yaegashi N., Hashimoto K. (2018). Association between family members and risk of postpartum depression in Japan: Does “who they live with” matter?—The Japan environment and Children’s study. Soc. Sci. Med..

[35] Godil A., Mallick M.S.A., Adam A.M., Haq A., Khetpal A., Afzal R., Salim M., Shahid N. (2017). Prevalence and severity of depression in a Pakistani population with at least one major chronic disease. J. Clin. Diagn. Res. JCDR.

[36] Hastert T.A., Ruterbusch J.J., Nair M., Noor M.I., Beebe-Dimmer J.L., Schwartz K., Baird T.E., Harper F.W., Thompson H., Schwartz A.G. (2020). Employment outcomes, financial burden, anxiety, and depression among caregivers of African American cancer survivors. JCO Oncol. Pract..

[37] Yang S.-Y., Wang J.-D., Chang J.-H. (2020). Occupational therapy to improve quality of life for colorectal cancer survivors: A randomized clinical trial. Supportive Care Cancer.

[38] Ross C.E., Van Willigen M. (1997). Education and the subjective quality of life. J. Health Soc. Behav..

[39] Lee J.J. (2005). An exploratory study on the quality of life of older Chinese people living alone in Hong Kong. Quality-of-Life Research in Chinese, Western and Global Contexts.

[40] Canbaz S., Sünter A.T., Dabak S., Pekşen Y. (2003). The prevalence of chronic diseases and quality of life in elderly people in Samsun. Turk. J. Med Sci..

[41] Fleming A.R., Fairweather J.S., Leahy M.J. (2013). Quality of life as a potential rehabilitation service outcome: The relationship between employment, quality of life, and other life areas. Rehabil. Couns. Bull..

[42] Marquandt D. (1980). You should standardize the predictor variables in your regression models. Discussion of: A critique of some ridge regression methods. J. Am. Stat. Assoc..

[43] Charmarkeh H. (2017). Seniors and technologies: Issues of inclusion and exclusion. Can. J. Commun..

[44] National Communications Commission Report on the Result of the 107 Radio and TV Market Survey. https://www.ncc.gov.tw/chinese/files/19012/4007_40972_190129_4.pdf.

[45] Chiou T.-J. (2009). An Investigation of the Reading Habits and the Reading Needs of Public Libraries of the Elderly in Taiwan. Natl. Taiwan Libr..

[46] Nie N.H., Hillygus D.S., Erbring L. (2002). Internet use, interpersonal relations, and sociability. The Internet in Everyday Life.

[47] Awick E.A., Ehlers D.K., Aguiñaga S., Daugherty A.M., Kramer A.F., McAuley E. (2017). Effects of a randomized exercise trial on physical activity, psychological distress and quality of life in older adults. Gen. Hosp. Psychiatry.

[48] Golestanifar S., DashtBozorgi Z. (2020). The Effectiveness of acceptance and commitment based therapy on depression, psychological health and life expectancy of the elderly with nonclinical depression. Aging Psychol..

[49] Şahin D.S., Özer Ö., Yanardağ M.Z. (2019). Perceived social support, quality of life and satisfaction with life in elderly people. Educ. Gerontol..

[50] Pan H., De Donder L., Dury S., Wang R., De Witte N., Verté D. (2019). Social participation among older adults in Belgium’s Flanders region: Exploring the roles of both new and old media usage. Inf. Commun. Soc..

[51] He T., Huang C., Li M., Zhou Y., Li S. (2020). Social participation of the elderly in China: The roles of conventional media, digital access and social media engagement. Telemat. Inform..

[52] Rashedi V., Gharib M., Yazdani A.A. (2014). Social participation and mental health among older adults in Iran. Iran. Rehabil. J..

[53] Billington J., Dowrick C., Hamer A., Robinson J., Williams C. (2010). An investigation into the therapeutic benefits of reading in relation to depression and well-being. Liverpool: The Reader Organization, Liverpool Health Inequalities Research Centre.

[54] DeWalt D.A., Pignone M.P. (2005). Reading is fundamental: The relationship between literacy and health. Arch. Intern. Med..

[55] Gaia A., Sala E., Cerati G. (2021). Social networking sites use and life satisfaction. A quantitative study on older people living in Europe. Eur. Soc..

[56] Millard A., Baldassar L., Wilding R. (2018). The significance of digital citizenship in the well-being of older migrants. Public Health.

